# Age at Puberty and the Emerging Obesity Epidemic

**DOI:** 10.1371/journal.pone.0008450

**Published:** 2009-12-24

**Authors:** Lise Aksglaede, Anders Juul, Lina W. Olsen, Thorkild I. A. Sørensen

**Affiliations:** 1 Department of Growth and Reproduction, University of Copenhagen, Copenhagen, Denmark; 2 Institute of Preventive Medicine, Centre for Health and Society, Copenhagen, Denmark; University of Córdoba, Spain

## Abstract

**Background:**

Recent studies have shown that puberty starts at younger ages than previously. It has been hypothesized that the increasing prevalence of childhood obesity is contributing to this trend. The purpose of this study was to analyze the association between prepubertal body mass index (BMI) and pubertal timing, as assessed by age at onset of pubertal growth spurt (OGS) and at peak height velocity (PHV), and the secular trend of pubertal timing given the prepubertal BMI.

**Methodology/Principal Findings:**

Annual measurements of height and weight were available in all children born from 1930 to 1969 who attended primary school in the Copenhagen municipality; 156,835 children fulfilled the criteria for determining age at OGS and PHV. The effect of prepubertal BMI at age seven on these markers of pubertal development within and between birth cohorts was analyzed. BMI at seven years was significantly inversely associated with age at OGS and PHV. Dividing the children into five levels of prepubertal BMI, we found a similar secular trend toward earlier maturation in all BMI groups.

**Conclusion/Significance:**

The heavier both boys and girls were at age seven, the earlier they entered puberty. Irrespective of level of BMI at age seven, there was a downward trend in the age at attaining puberty in both boys and girls, which suggests that the obesity epidemic is not solely responsible for the trend.

## Introduction

From the late 19^th^ century to the mid 20^th^ century a gradual decline in age at puberty has been reported in girls, after which this trend ceased most likely as a result of increased stability in socio-economic conditions, nutritional status and hygiene [1]. However, we recently reported a significant decline in age at pubertal maturation in Danish children born in 1930 through 1969 [2], and since the beginning of the 1990'ies further evidence of a shift towards earlier maturation in US [3]–[7] and Danish [8], [9] children compared with previous studies [10]–[15] has been observed. Nevertheless, the validity of the data on US boys has been debated, and an expert panel has recently concluded that, the currently available data are insufficient in quality and quantity to confirm a change in pubertal timing in US boys [16].

Puberty is the end-point of a complex series of developmental events, and identification of the trigger(s) of pubertal onset has drawn considerable attention. The pubertal cascade is initiated by the activation of the hypothalamic-pituitary-gonadal axis leading to the development of secondary sexual characteristics; however, to date no specific factor responsible for this activation has been identified. The ‘critical weight’ hypothesis suggested by Frisch and Revelle [17], [18] in the early 1970'ies proposed a minimum weight of 48 kg or 22% body fat to allow puberty to start. The recent secular trends in pubertal maturation seem to coincide with the increasing prevalence of overweight and obesity [19]–[21],) and have raised considerable discussion as to whether the early maturation is due to the obesity epidemic.

In Denmark data from school health records including height and weight from 350,445 boys and girls born in 1930 through 1983 has been entered into the computerized Copenhagen School Health Records Register [22]. Although no major changes over time in the central percentiles of the BMI distribution have been found, there has been a marked increase in the prevalence of overweight and obesity in this cohort [23]–[26]. In addition, we found a significant decline in the age at pubertal maturation as assessed by age at onset of pubertal growth spurt (OGS) and age at peak height velocity (PHV) in a subgroup of 156,835 girls and boys born in 1930 through 1969 from the same cohort [2]. We therefore aimed at analyzing the association between prepubertal BMI at 7 years and pubertal timing at the individual level in this large cohort to determine if there was any association between the degree of prepubertal overweight/obesity and age at sexual maturation, and if age at sexual maturation declined irrespectively of the degree of overweight/obesity.

## Methods

### Participants

All children born 1930 through 1969 who attended private or public primary schools in the Copenhagen Municipality underwent regular school health examinations. These were carried out annually and were mandatory until July 1984. The school health records included measurements of height, weight, and time of measurement (year and month). Height and weight were measured to the nearest 1 cm and the nearest 100 grams, respectively. Throughout the entire period, measurements were taken with children wearing minimal clothing and no shoes. Information from these records has been entered into the computerized Copenhagen School Health Records Register [22] and used for estimating age at onset of pubertal growth spurt (OGS) and at peak height velocity (PHV) as markers for pubertal maturation. Subjects eligible for the study were children born 1930 through 1969, who had a minimum of five height measurements recorded on the school health record. We further limited the analyzis to girls who had at least one height measurement recorded in their thirteenth year or later, and to boys who had a height measurement recorded in their fifteenth year or later.

### Measures

Height velocity was defined as the difference in heights at two adjacent measurements, divided by the difference in time, and this value was assigned at an age in the middle of this period. Age at OGS was defined as the ‘latest’ minimum before age at PHV. Age at PHV was defined as the age at maximal height velocity after the age of 8.0 years in girls, and after the age of 10.0 years in boys. Further details and criteria for estimating age at OGS and at PHV have been described previously [2].

Body mass index (BMI) was calculated as weight/height^2^ (kg/m^2^) for each child at age 7 years to ensure a prepubertal estimate. Using data from health examinations performed between 1955 and 1960 internal age- and sex-specific BMI references were created [27]. Data from these years were used because the prevalence of overweight was low and stable during this period. BMI z-scores were calculated by subtracting the BMI for each child from the mean BMI in the fixed reference population, and dividing the result by the standard deviation in the reference population. If two measurements of BMI were available before and after age 7 years, the SD score was interpolated to the age of 7.0 years. Otherwise, if a BMI measurement existed within 7.0 years plus/minus 12 months this measurement was used. Positive values indicate BMI values above the average in the reference population, and negative values indicate BMI values below the average. Children were divided into 5 categories according to BMI SDS: <−0.75, −0.75 to −0.25, −0.25 to +0.25, +0.25 to +0.75 and >+0.75.

### Statistical Analysis

The effect of BMI at age 7 years on age at pubertal growth spurt was analyzed using regression analyzis with age at OGS and age at PHV, respectively, as dependent variables and the combinations of categories of BMI SDS at age 7 years and time categorized into 5-year categories as explanatory variables. The two types of censoring; left censoring (if the child had not reached puberty before leaving school) and interval censoring (if the child had stopped growing when leaving school, but the yearly measurement left us unable to determine the age at onset or peak), were taken into account by using SAS' proc lifereg, which fits an ordinary regression model, in the present setting with normal distributed error terms, to failure time data that can be right, left or interval censored.

### Ethics

Since the study is entirely based on register data, there was according to Danish law no request for an ethical permission. The Danish Data Protection Agency approved the study.

## Results

156,835 children (21,612 boys; and 135,223 girls) fulfilled the criteria for determining age at OGS and PHV as previously described [2].

**Figure 1:**
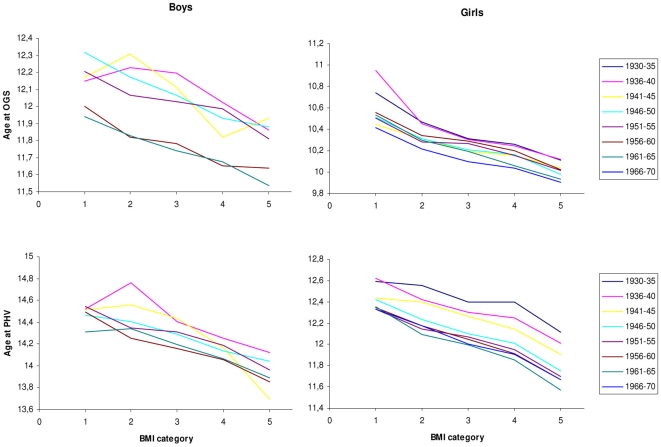
Age at OGS and PHV according to BMI. Age at OGS (upper panel) and at PHV (lower panel) according to BMI category 1–5 divided by birth cohort. BMI category 1, 2, 3, 4 and 5 = BMI SDS: <−0.75, −0.75 to −0.25, −0.25 to +0.25, +0.25 to +0.75 and >+0.75, respectively. Note data in boys only shown from birth year 1940–69 due to a small number of samples before that time.

In all birth cohorts in both girls and boys prepubertal BMI was significantly inversely associated with earlier age at OGS and PHV, although with some irregularity in boys from the earliest birth cohorts (Figure 1).

**Figure 2:**
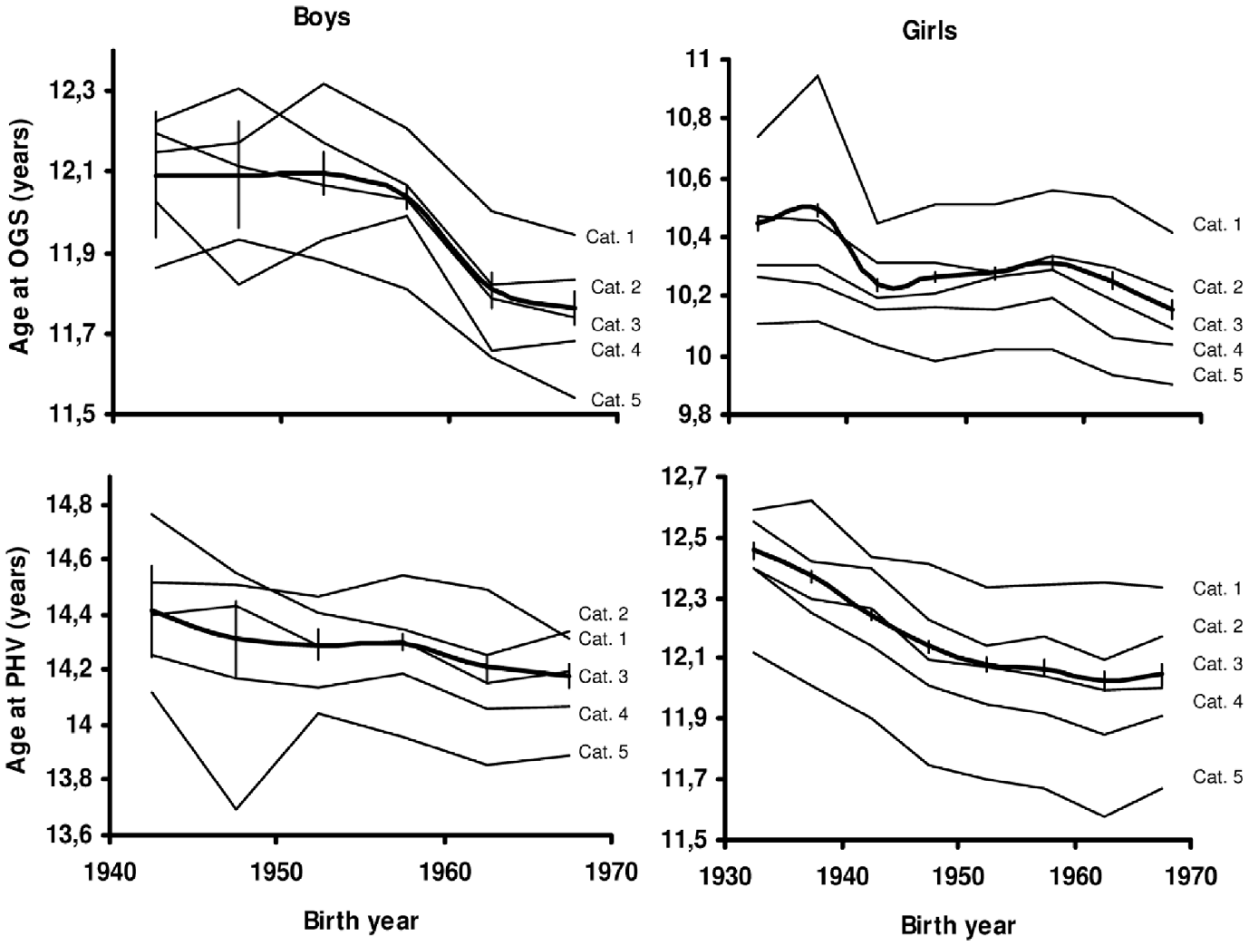
Age at OGS and PHV according to birth year. Age at OGS (upper panel) and at PHV (lower panel) according to year of birth. Wide lines represent data on the whole cohort including confidence intervals, whereas the slimmer lines represent data on children according to BMI category 1–5. BMI category 1, 2, 3, 4 and 5 = BMI SDS: <−0.75, −0.75 to −0.25, −0.25 to +0.25, +0.25 to +0.75 and >+0.75, respectively. Note data in boys only shown from birth year 1940–69 due to a small number of samples before that time.

Figure 2 shows the secular trend in OGS and PHV in the entire cohort (as previously reported [2]), and superimposed on these curves, the secular trends for each of the five levels of BMI at age 7 years. The downward secular trend towards earlier maturation estimated by OGS as well as PHV was evident in all BMI categories in both genders, though with a less regular course for the boys than for the girls, most likely because of the much smaller sample size of boys.

The range of differences in OGS and PHV between the lower and upper quintiles of BMI and the changes observed in OGS and PHV by birth cohort are presented in Table S1. This table shows that the magnitudes of differences by degree of obesity are greater than the magnitude of changes over time.

## Discussion

In this large population-based cohort study of 156,835 children we found a significant effect of BMI at 7 years on age at sexual maturation in both boys and girls; the heavier at age 7 years the earlier did the children enter puberty whether assessed by OGS or PHV. We found a downward secular trend in the age at attaining puberty irrespective of size of BMI at age 7 in both boys and girls, corresponding to the decline reported earlier for the whole group [2], clearly indicating that the decline cannot be attributed to increasing degree of obesity in this population.

The association between body weight and timing of sexual development has been reported previously. However, most such studies are based on cross-sectional data [28]–[36], and the possible causal sequence of the association between body fat and sexual maturation therefore is not clearly elucidated in these studies. However, one population-based growth study on 3650 Swedish boys and girls born in the early 1970s who were followed from birth to 18 years of age, found a significant association between BMI gain between 2 and 8 years and the age at attaining PHV, i.e. a larger gain in prepubertal BMI was associated with an earlier onset of puberty [37]. Mamun *et al* found that increasing BMI and overweight at age 5 years in 2897 boys and girls was related to advanced age at pubertal maturation at 14 years of age as assessed by self reporting [38]. Another longitudinal study on 183 girls who were examined at age 5, 7 and 9 years found that girls with higher weight at 5 years of age were more likely to exhibit more advanced pubertal development at age 9 years [39]. Likewise, Lee *et al* found a higher BMI at age 36 months and a higher rate of change in BMI during childhood to be associated with an earlier puberty in girls [40].

The majority of studies on the timing of puberty report only on changes in girls, and most often the age at menarche has been used as a marker for the timing, because it is easily accessible by questioning the girls, and such self-reported data are relatively reliable [41], [42]. In contrast, in boys no such characteristic event in puberty exists, and clinical examination of boys is usually required for such assessment, although, one study reported on age at voice break and its relation to prepubertal BMI [8]. Therefore, data on the relation between body weight and timing of puberty in boys are contradicting. However, most studies, like ours, report an association between BMI and earlier age at pubertal maturation [8], [28], [37], [38], [43], [44], whereas others find the reverse association [34].

Studies using timing of the pubertal growth as a marker are very rare, because they need longitudinal measurements of height and are therefore time consuming [2], [37], [43]–[45]. In a study on 99 monozygotic and 76 dizygotic male twin pairs, Silventoinen *et al* showed that increasing childhood BMI was correlated with an earlier pubertal growth spurt, and that a strong genetic factor behind this association existed. Thus, growth during puberty was strictly genetically regulated, and these genetic factors also explained why boys who matured early had higher BMI through childhood in this study [44]. Interestingly, Buyken *et al* found that BMI and fat mass/m^2^ one and two years before the onset of pubertal growth spurt was associated with late pubertal markers, such as age at PHV in both sexes and age at menarche in girls, but not with the *onset* of growth spurt. These results contrast our finding of an equal association between OGS and PHV and prepubertal BMI in both girls and boys. However, Silventoinen confirms our findings in boys [44], whereas the remaining studies on timing of growth spurt only report on PHV [37], [43].

The role of body weight in the complex developmental process has drawn considerable attention since the ‘critical weight’ hypothesis was suggested by Frisch and Revelle [17], [18]. Later, the link between body fat and puberty was supported by the discovery of the adipocyte-derived hormone, leptin [46]. Leptin is secreted from the fat tissue in direct proportion to the amount of total fat mass, and signals the state of energy reserves through a hypothalamic receptor to regulate appetite and reproductive function; acting as an essential permissive factor for the onset of puberty [47]. Recent studies have suggested that the mechanism whereby leptin positively modulates puberty onset may include the Kiss1/Kiss1R system (for review see [48]). Kisspeptin regulates the hypothalamic-pituitary-gonadal axis directly via gonadotropin releasing hormone neurons and during the last 5 years KiSS-1 has been substantiated as a pivotal regulator of puberty in mammals.

Increases in body fat may directly affect the hormonal regulation of puberty; increased aromatase activity, and thereby increased conversion of androgens to estrogen may promote earlier breast development [49]. Additionally, in the presence of insulin resistance, compensatory hyperinsulinaemia usually results in reduced levels of sex hormone binding globulin [50]. Thus, the bioavailability of sex steroids is potentially increased in obese insulin-resistant children.

We find that a higher prepubertal BMI is associated with an earlier pubertal growth spurt, but trends in BMI do not independently explain the secular trend during the study period. Thus, we observed an almost parallel decline in age at OGS and PHV in all BMI quintiles over time. Moreover, as mentioned in the introduction no corresponding changes in the central part of the BMI distribution have been observed in the same study population.

Endocrine disrupting chemicals from the environment has been shown to influence puberty timing in animal studies as well as in wildlife observations [51], but little is known about the possible role of these chemicals in the timing and progression of puberty in humans. However, children and adolescents have been exposed to a large and increasing number of chemicals with endocrine disrupting properties during recent decades, and these have been demonstrated in fluids and tissues from children and adolescents [52]–[54]. It can be speculated that the children are exposed to different types and amounts of chemicals during the study period, which may account for this change in timing of pubertal growth spurt.

The uniqueness of our study is that it is a multi-cohort longitudinal study reporting data on all Copenhagen school children born in 1930 (boys 1935) through 1969 including yearly anthropometric measurements in each child from all years of attending primary school giving the opportunity to not only look at the association between prepubertal weight and sexual maturation but also on the association between weight and the secular trend in pubertal timing over four decades. The main strength of our study is that the school health examinations were mandatory and performed in all public and private schools during the study period, and the potential risk of selection bias (obese and/or early-developed children declining to participate) may thereby be considered very small. The extremes of obesity and leanness are thus represented in our data. Furthermore, our assessment of BMI at age 7 years insures a prepubertal BMI that is not affected by the increases in fat and weight gain related to puberty.

However, our data is limited by the relatively small number of boys fulfilling the criteria for determining age at OGS and PHV compared to girls, as previously described [2]. The number of children included in our study was reduced from 149,992 girls to 135,223, and 151,604 boys to 21,612, respectively, after application of our mathematical criteria for determining age at OGS and at PHV. The reason for this difference between the sexes is that school attendance was only mandatory for 7 years until 1972 in Denmark. One of our criteria was measurement in each girl after the age of 13 years and after the age of 15 years in boys, and many boys, but not girls, thus left school before this age. It may be speculated that the high proportion of growth charts from boys in which OGS and PHV could not be validly determined by our method could pose a theoretical selection bias. However, this may not affect the results presented, which all are based on strata of BMI at age 7 years, and differences in numbers of children within the strata due to such selection mechanism cannot bias the reported associations, neither within or between birth cohorts. Moreover, it is reassuring that the secular trends within each stratum of BMI at age 7 were similar to the overall trend in OGS and PHV.

In conclusion, during the study period the heaviest category of prepubertal children entered puberty significantly earlier than the lightest category of children. The combination of various hormonal stimuli related to increased body fat; increased conversion of androgens to estrogen and increased bioavailability of circulating sex steroids could contribute to earlier activation of the hypothalamic-pituitary-gonadal axis and thereby to the earlier initiation of puberty in the obese. However, the secular change was found in all BMI categories suggesting that the obesity epidemic is not solely responsible.

## Supporting Information

Table S1 The range of differences in OGS and PHV between the lower and upper quintiles of BMI and the changes observed in OGS and PHV by birth cohort. Data presented as delta age (months). nd = no data.
(0.02 MB DOC)
