# Supplementary material for: Age at Puberty and the Emerging Obesity Epidemic
Source: PLoS One. 2009 Dec 24;4(12):e8450. doi: 10.1371/journal.pone.0008450 (PMC2793517; doi:10.1371/journal.pone.0008450)
Supplement: Supplementary file 1 [file pone.0008450.s001.doc]

Table S1

	Girls				Boys			
	OGS		PHV		OGS		PHV	
	BMI	Birth cohort	BMI	Birth cohort	BMI	Birth cohort	BMI	Birth cohort
1930-1935	-7,6	0,0	-5,7	0,0	nd	nd	nd	nd
1936-1940	-10,0	0,5	-7,4	-1,1	nd	nd	nd	nd
1941-1945	-4,9	-2,5	-6,4	-2,6	-3,4	0,0	-4,8	0,0
1946-1950	-6,4	-2,2	-8,0	-3,8	-2,9	0,0	-9,8	-1,2
1951-1955	-5,9	-2,0	-7,6	-4,6	-5,2	0,0	-5,0	-1,5
1956-1960	-6,4	-1,6	-8,1	-4,7	-4,7	-0,7	-7,0	-1,3
1961-1965	-7,2	-2,3	-9,3	-5,2	-4,3	-3,4	-7,7	-2,4
1966-1969	-6,2	-3,5	-8,0	-5,0	-4,8	-4,0	-5,0	-2,8
